# Ventilation-to-perfusion relationships and right-to-left shunt during neonatal intensive care in infants with congenital diaphragmatic hernia

**DOI:** 10.1038/s41390-022-02001-2

**Published:** 2022-03-19

**Authors:** Theodore Dassios, Fahad M. Shareef Arattu Thodika, Emma Williams, Mark Davenport, Kypros H. Nicolaides, Anne Greenough

**Affiliations:** 1 Neonatal Intensive Care Centre, King’s College Hospital NHS Foundation Trust, London, UK; 2 Women and Children’s Health, School of Life Course Sciences, Faculty of Life Sciences and Medicine, King’s College London, London, UK; 3 Department of Paediatric Surgery, King’s College Hospital NHS Foundation Trust, London, UK; 4 Harris Birthright Research Centre for Fetal Medicine, King’s College London, London, UK; 5 MRC-Asthma UK Centre in Allergic Mechanisms of Asthma, King’s College London, London, UK; 6 National Institute for Health Research (NIHR) Biomedical Research Centre based at Guy’s and St Thomas’ NHS Foundation Trust and King’s College London, London, UK

## Abstract

**Background:**

We aimed to explore the postnatal evolution of ventilation/perfusion ratio (*V*_A_/*Q*) and right-to-left shunt in infants with congenital diaphragmatic hernia (CDH) and whether these indices predicted survival to discharge.

**Methods:**

Retrospective cohort study at King’s College Hospital, London, UK of infants admitted with CDH in 10 years (2011–2021). The non-invasive method of the oxyhaemoglobin dissociation curve was used to determine the *V*_A_/*Q* and shunt in the first 24 h of life, pre-operation, pre-extubation and in the deceased infants, before death.

**Results:**

Eighty-two infants with CDH (71 left-sided) were included with a median (IQR) gestation of 38.1(34.8–39.0) weeks. Fifty-three (65%) survived to discharge from neonatal care. The median (IQR) *V*_A_/*Q* in the first 24 h was lower in the deceased infants [0.09(0.07–0.12)] compared to the ones who survived [0.28(0.19–0.38), *p* < 0.001]. In the infants who survived, the *V*_A_/*Q* was lower in the first 24 h [0.28 (0.19–0.38)] compared to pre-operation [0.41 (0.3–0.49), *p* < 0.001] and lower pre-operation compared to pre-extubation [0.48 (0.39–0.55), *p* = 0.027]. The shunt was not different in infants who survived compared to the infants who did not.

**Conclusions:**

Ventilation-to-perfusion ratio was lower in infants who died in the neonatal period compared to the ones that survived and improved in surviving infants over the immediate postnatal period.

**Impact:**

- The non-invasive method of the oxyhaemoglobin dissociation curve was used to determine the ventilation/perfusion ratio *V*_A_/*Q* in infants with congenital diaphragmatic hernia (CDH) in the first 24 h of life, pre-operation, pre-extubation and in the deceased infants, before death.
- The *V*_A_/*Q* in the first 24 h of life was lower in the infants who did not survive to discharge from neonatal care compared to the ones who survived.
- In the infants who survived, the *V*_A_/*Q* improved over the immediate postnatal period.
- The non-invasive calculation of *V*_A_/*Q* can provide valuable information relating to survival to discharge.

## Introduction

Congenital diaphragmatic hernia (CDH) is associated with high mortality and respiratory morbidity lasting into childhood and early adulthood.^1, 2^ Pulmonary hypoplasia is a major determinant of outcomes in infants with CDH.^3, 4^ Pulmonary vascular structural abnormalities are also well described in CDH with a smaller number of arteries and increased muscular wall thickness.^5^ Histology and three-dimensional reconstruction have identified prominent bronchopulmonary vascular anastomoses in the lungs of infants who died with severe CDH, with this abnormal development likely representing evidence of intrapulmonary shunting and contributing to the refractory hypoxaemia seen in the severe form of the disease.^6^

The severity of hypoxaemia is determined by the relationship of ventilation to perfusion (*V*_A_/*Q*) and the extent of intrapulmonary shunting.^7^ Although *V*_A_/*Q* abnormalities have been reported in surviving CDH children longitudinally,^8^ these abnormalities have never been explored in the immediate postnatal period, or in infants who did not survive to discharge from neonatal care. The highly specialised and invasive nature of nuclear medicine and histological/post-mortem methods, partly explain this lack of early data in CDH infants. We have recently applied an alternative, non-invasive method to assess the *V*_A_/*Q* and intrapulmonary shunt using the oxyhaemoglobin dissociation curve (ODC) and reported normal values in healthy term infants.^9, 10^ More so, we described abnormally low *V*_A_/*Q* and high shunt in preterm infants with evolving respiratory disease^11^ as well as in older children with hepatopulmonary syndrome.^12^ This method could thus be further utilised to describe oxygenation abnormalities in CDH, as it non-invasively quantifies *V*_A_/*Q* mismatch and right-to-left shunt, both of which are contributing factors to the underlying hypoxaemia in CDH.

We aimed to use the non-invasive ODC method to describe the evolution of *V*_A_/*Q* and shunt abnormalities in infants with CDH during their neonatal stay and compare those early abnormalities in infants who survived to discharge from neonatal care compared to those seen in infants who did not survive.

## Methods

### Subjects

Newborn infants treated for CDH at King’s College Hospital (KCH) Foundation Trust, London, UK during a 10-year period (1 January 2011–1 January 2021) were included in the study. KCH is a tertiary referral centre for infants with CDH who might benefit from foetal endoscopic tracheal occlusion (FETO); hence, our population includes infants at the severe end of the disease spectrum. The indications for FETO have been previously described.^13^ The infants were intubated at birth and were ventilated on conventional mechanical ventilation. If the pre-ductal transcutaneous oxygen saturation was below 90% while ventilated with a peak inflation pressure >30 cm H_2_O the infants were then ventilated with high-frequency oscillation (HFO). Infants were referred for extracorporeal membrane oxygenation (ECMO) following discussion with an ECMO centre, if the echocardiographic assessment was suggestive of significant pulmonary hypertension, which is theoretically a reversible condition.^14^The infants were deemed stable to undergo an operation when they did not require inhaled nitric oxide (NO), were requiring a fraction of inspired oxygen (F_I_O_2_) < 0.50 and were on minimal inotropic support (one inotropic agent, usually dopamine infusion of 5–10 mcg/kg/min). The study was registered as a service evaluation with the Clinical Governance Department of KCH. The Health Research Authority Toolkit of the National Health System, United Kingdom confirmed that the study would not be considered as research and would not require regulatory approval by a research ethics committee.

### Calculation of *V*_A_/*Q* and shunt with the oxygen dissociation curve

**Fig. 1:**
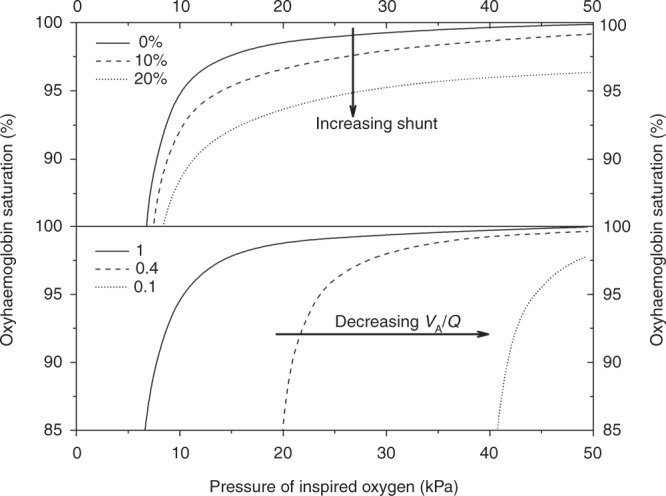
The oxyhaemoglobin dissociation curve: proportion of haemoglobin saturation versus pressure of inspired oxygen. Increasing shunt (upper diagram) displaces the curve downwards and decreasing *V*_A_/*Q* (lower diagram) shifts the curve to the right. The dashed line in the upper diagram represents a subject with a shunt of 10% and the dotted line a subject with a shunt of 20%. The dashed line in the lower diagram represents a subject with a *V*_A_/Q of 0.4 and the dotted line a subject with a *V*_A_/*Q* of 0.1. Reproduced from Dassios et al.^11^

The method has been previously described in detail.^11^ The relative position of an individual’s oxyhaemoglobin dissociation curve can be used to calculate the degree of shunt and *V*_A_/*Q* inequality as a non-invasive measure of oxygenation impairment.^9^ Briefly, right-to-left shunt causes a decrease in arterial oxygen saturation, which cannot be overcome by increasing the amount of inspired oxygen. The level of the right-to-left shunt can be thus quantified by the degree of the depression of the oxyhaemoglobin dissociation curve. A reduction in the *V*_A_/*Q* results in a post-alveolar reduction in the blood oxygen content, which produces a rightward shift of the curve. This shift can be overcome by increasing the fraction of inspired oxygen. The reduction, thus, in *V*_A_/*Q* can be measured by the degree of the right shift of the curve compared to an ideal reference curve (Fig. 1). Similarly, the further downwards an individual’s curve is positioned relative to the ideal curve, the higher that individual’s right-to-left shunt (Fig. 1).^15^ Using at least two paired values of peripheral oxygen saturation (SpO_2_) and F_I_O_2_ for each infant taken ≤4 h apart, an oxyhaemoglobin dissociation curve was constructed for each subject. Using the paired values of F_I_O_2_ and SpO_2_, the *V*_A_/*Q* and the percentage of right-to-left shunt were calculated at four time endpoints: during the first 24 h of life, in the 12 h before operation (pre-operation), in the 12 h before extubation (pre-extubation) and, in the infants who died, in the last 12 h before death.^15^ *V*_A_/*Q* and right-to-left shunt were derived using software that derives results for each dataset from a two-compartment model: shunt and *V*_A_/*Q* for a single homogeneous ventilated compartment.^16^ The contemporaneous haemoglobin value from the arterial blood gas analysis at the time of each assessment was used in the calculations. For ventilated infants treated with NO, the F_I_O_2_ was measured and recorded as delivered to the patient from the NO delivery system.

### Information from the medical notes

The following information was collected from the medical and surgical notes: observed-to-expected lung-to-head ratio (LHR) at diagnosis,^17^ FETO procedure (yes/no),^18^ sex, gestational age (weeks), birth weight (kg), Apgar score at 5 min, left- or right-sided defect, intraoperative confirmation of the liver above the diaphragm (liver up), operated (yes/no), age at surgery (days), pre-operative use of HFO (yes/no), duration of mechanical ventilation (days), preoperative use of NO (yes/no), duration of stay (days), ECMO (yes/no), survival to discharge from neonatal care (yes/no).

### Statistics

Data were tested for normality with the Kolmogorov–Smirnov test and found to be non-normally distributed and hence presented as median and interquartile (IQR) range. Differences in the *V*_A_/*Q* and right-to-left shunt were compared at the different time endpoints using the Wilcoxon signed-rank test. The relationship of *V*_A_/*Q* in the first 24 h of life with LHR, duration of mechanical ventilation, and total duration of stay were examined in the infants who survived with the Kendall-tau rank correlation coefficient. Differences in *V*_A_/*Q*, shunt and clinical parameters between infants who survived to discharge from neonatal care and deceased infants were assessed for statistical significance using the Mann–Whitney rank-sum test or Chi-squared test, as appropriate. The factors that were statistically different (*p* value <0.05) were inserted into a multivariable binary regression model with survival to discharge from neonatal care as the outcome variable. Variables without normal distribution were logarithmically transformed. Multi-collinearity among the independent variables in the regression analysis was assessed by the calculation of the tolerance for the independent variables. The performance of *V*_A_/*Q* in predicting survival to discharge from neonatal care was assessed by receiver operator characteristic (ROC) curve analysis and estimation of the corresponding area under the curve (AUC). One optimal cut-point from the ROC curve was selected to report the corresponding sensitivity and specificity. The cut point was selected on the basis of the optimal combination for the highest values of both sensitivity and specificity.

Statistical analysis was performed using SPSS software (SPSS Inc., Chicago, IL).

## Results

During the study period, 104 infants with CDH were treated at the neonatal unit at KCH. Thirteen infants were excluded because of a lack of complete medical and nursing notes. Nine infants were excluded as they were nursed in a F_I_O_2_ of 1.0 for the whole duration of their stay and we were thus unable to derive paired values of SpO_2_ and F_I_O_2_. These nine infants all died because of hypoxaemic respiratory failure within the first 24 h of life before they could have surgery. Eighty-two (45 male) infants with a median (IQR) gestational age of 38.1 (34.8–39.0) weeks were included in the study. They had a median (IQR) birth weight of 2.70 (2.23–3.16) kg. The antenatal, demographic, surgical and neonatal characteristics of the included infants are presented in Table 1. The median (IQR) *V*_A_/*Q* on the first day after birth was 0.20 (0.09–0.32) and the shunt was 10 (3–18)%.

**Table 1:** Characteristics of the study population.

	Number of infants	82
Antenatal	Lung to head ratio (%)	24 (14–36)
	Antenatal FETO procedure	28 (34)
Demographics and birth	Male sex	45 (55)
	Gestation (weeks)	38.1 (34.8–39.0)
	Birth weight (kg)	2.70 (2.23–3.16)
	Apgar score at 5 min	8 (6–9)
Surgical	Left-sided hernia	71 (87)
	Liver up	13 (16)
	Operated	54 (66)
	Days to surgery (for the operated)	4 (3–6)
Neonatal	HFO	41 (50)
	Days of mechanical ventilation	6 (3–10)
	Nitric oxide	54 (66)
	Duration of stay (days)	18 (2–31)
	Survived to discharge from neonatal care	53 (65)

Data are presented as median (interquartile range) or number (percentage).

**Fig. 2:**
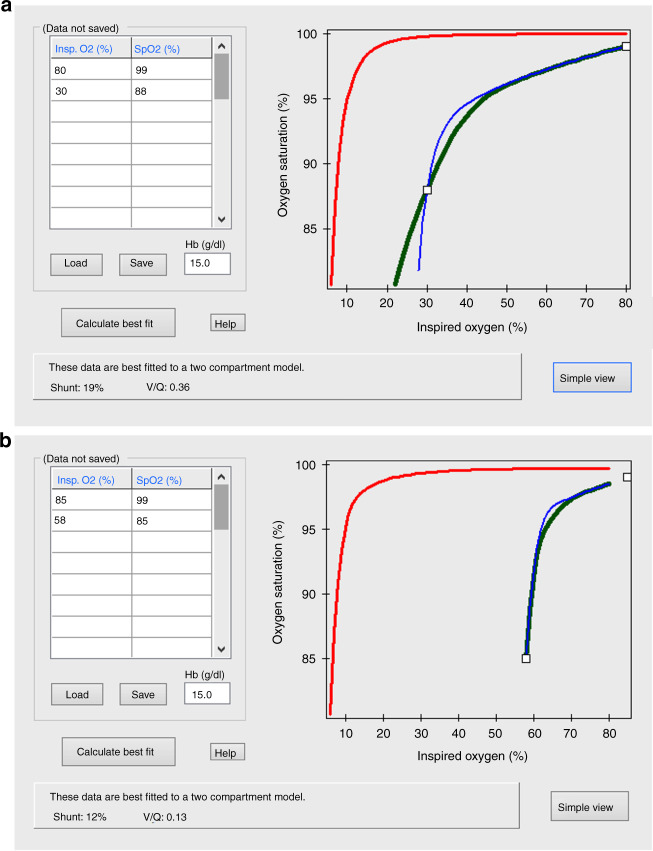
Survival to discharge from neonatal care. Representative oxyhaemoglobin dissociation curves of an infant who survived (**a**) and an infant who did not survive (**b**). The paired values of F_I_O_2_ and SpO_2_ that were used for the calculations of *V*_A_/*Q* and shunt are presented in the nested table on the left hand side of the figure. The contemporaneous values of the haemoglobin (Hb) concentration are also presented.

Fifty-three infants (65%) survived to discharge from neonatal care. The median (IQR) duration of stay in the deceased infants was 2 (1–3) days. The cause of death in all deceased infants was hypoxic respiratory failure secondary to the CDH. The infants who survived had a higher median (IQR) Apgar score at 5 min [8 (7–9)] compared to the infants who did not survive [6 (3–8), *p* = 0.001] and were less frequently treated with HFO and NO (26 and 43% compared to 90 and 100%, respectively, *p* < 0.001 for both, Table 2). The infants who survived had a higher median (IQR) *V*_A_/*Q* in the first 24 h [0.28 (0.19–0.37)] compared to the infants who did not survive [0.09 (0.07–0.12), *p* < 0.001, Fig. 2]. The shunt in the first 24 h was not different in the infants who survived [9 (4–16)%] compared to the infants who did not survive [11 (2–20)%, *p* = 0.646, Fig. 2]. Three infants had ECMO and all three died in the ECMO referral centre before they could have surgery. Their *V*_A_/*Q* ratios in the first 24 h of life were 0.07, 0.08 and 0.09.

**Table 2:** Comparison of demographics, respiratory parameters, *V*_A_/*Q* and shunt in infants who survived to discharge compared to those who did not.

	Survived	Deceased	*p* value
Number of infants	53 (65)	29 (35)	N/A
LHR (%)	23 (11–33)	23 (14–35)	0.552
Antenatal FETO procedure	18 (34)	10 (34)	0.948
Male sex	29 (55)	15 (52)	0.486
Gestation (weeks)	38.1 (35.2–39.1)	36.8 (34.2–39.0)	0.308
Birth weight (kg)	2.77 (2.26–3.22)	2.65 (1.94–2.97)	0.181
Apgar score at 5 min	8 (7–9)	6 (3–8)	0.001
Left-sided hernia	43 (81)	26 (90)	0.933
Liver up	9 (17)	4 (14)	0.555
HFO	14 (26)	26 (90)	<0.001
Nitric oxide	23 (43)	29 (100)	<0.001
*V*_A_/*Q* in the first 24 h	0.28 (0.19–0.37)	0.09 (0.07–0.12)	<0.001
Shunt (%) in the first 24 h	9 (4–16)	11 (2–20)	0.646

Data are presented as median (interquartile range) or number (percentage). Mann– Whitney *U* or Chi-square test as appropriate.

*LHR* lung-to-head ratio, *FETO* foetal endoscopic tracheal occlusion, *HFO* high-frequency oscillation, *N/A* not applicable.

In the infants who survived, *V*_A_/*Q* correlated with duration of mechanical ventilation (*r* = −0.377, *p* < 0.001) and the total duration of stay (*r* = -0.279, *p* = 0.004) but not with the LHR at diagnosis (*r* = 0.114, *p* = 0.349).

**Fig. 3:**
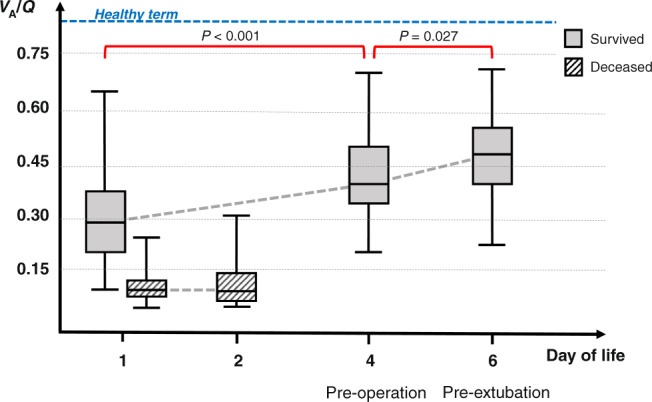
Ventilation-to-perfusion ratios in infants with CDH who survived to discharge and infants who did not. The median duration of stay for the deceased infants was 2 days. The surviving infants were operated at a median age of 4 days and were extubated at a median age of 6 days. A reference line with the mean *V*_A_/*Q* for healthy term infants is also depicted.

The evolution of *V*_A_/*Q* in the infants who survived and in the infants who did not survive is presented in Fig. 3. In the infants who survived the median (IQR) *V*_A_/*Q* was lower in the first 24 h [0.28 (0.19–0.38)] compared to pre-operation [0.41 (0.34–0.49), *p* < 0.001] and lower pre-operation compared to pre-extubation [0.48 (0.39–0.55), *p* = 0.027]. In the infants who survived, the shunt was not different in the first 24 h [9 (4–16)%] compared to pre-operation [7 (0–13)%, *p* = 0.064] and not different pre-operation compared to pre-extubation [1 (0–10)%, *p* = 0.058]. In the infants who did not survive, the median (IQR) *V*_A_/*Q* [0.09 (0.08–0.12)] and shunt [11 (0–20)%] in the first 24 h were not different compared to the *V*_A_/*Q* [0.08 (0.07–0.14), *p* = 0.398] and shunt [4 (0–19)%, *p* = 0.337] before they died.

Twenty-eight infants (34%) underwent the antenatal FETO procedure. The median (IQR) *V*_A_/*Q* in the first 24 h was not different in infants who underwent the FETO procedure [0.13 (0.09–0.24)] compared to the infants who did not [0.20 (0.09–0.33), *p* = 0.252]. The median (IQR) shunt in the first 24 h was not different in infants who underwent the FETO procedure [12 (2–22)%] compared to the infants who did not [9 (1–17)%, *p* = 0.474].

**Fig. 4:**
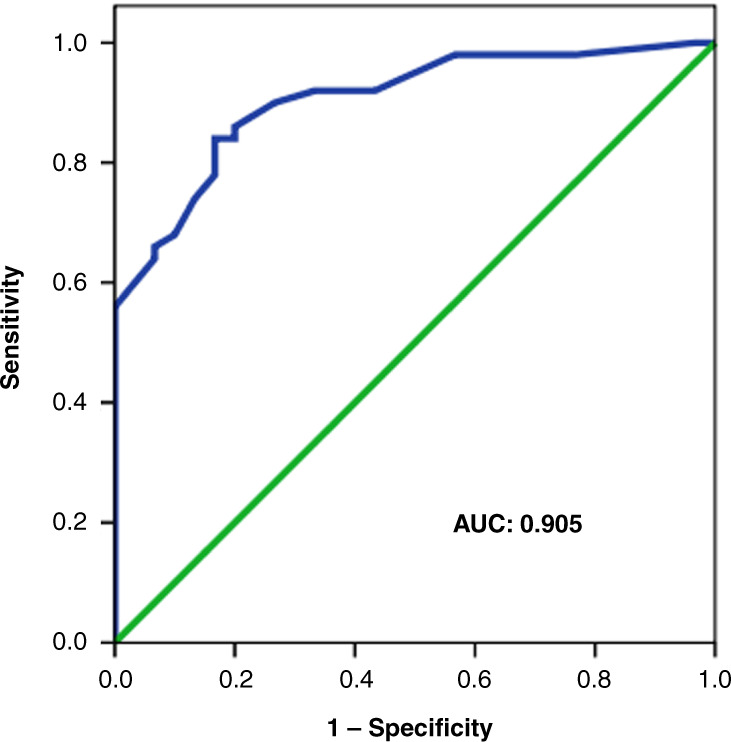
Receiver operator characteristic curve analysis and estimation of the corresponding area under the curve (AUC) for the performance of *V*_A_/*Q* in the first 24 h of life in predicting survival to discharge from neonatal care.

Following multivariable binary regression analysis with survival to discharge as the outcome variable, *V*_A_/*Q* (adjusted *p* = 0.001) and high-frequency ventilation (adjusted *p* = 0.003) were significantly related to survival, while Apgar score at 5 min (*p* = 0.064) was not related to survival. The receiver operator characteristic curve analysis demonstrated that in predicting survival to discharge, the *V*_A_/*Q* in the first 24 h had an AUC of 0.905. A *V*_A_/*Q* in the first 24 h of life >0.15 predicted survival to discharge from neonatal care with 84% sensitivity and 88% specificity (Fig. 4).

## Discussion

We have demonstrated that in surviving infants with CDH, the relationship of ventilation to perfusion gradually improved over the immediate postnatal period. The infants who did not survive started with severely abnormal ventilation-perfusion relationships, which remained abnormally low, up to their death. We also demonstrated that the ventilation-perfusion ratio in the first day of life predicted mortality in CDH infants with moderate–high sensitivity and specificity. The shunt was not significantly different between infants who survived and infants who did not survive and did not change significantly in the infants who survived during the immediate postnatal period.

Few previous studies have assessed ventilation to perfusion relationships in infants with CDH. Björkman et al. used single-photon emission computed tomography in twelve CDH infants at a mean age of six months and reported varying degrees of ventilation/perfusion abnormalities, which correlated with the presence of pulmonary artery hypertension and duration of ventilation. They concluded that pulmonary hypertension appears to be an important pathophysiological factor related to ventilation/perfusion abnormalities, and that pulmonary hypertension persists into the postoperative neonatal period.^19^ Similarly, Dao et al. tracked the *V*_A_/*Q* measured by serial nuclear medicine scans in 212 survivors of CDH over 15 years and reported a supra-physiological increase of the average ipsilateral *V*_A_/*Q* over time which was driven by a progressive reduction in perfusion.^8^ The finding of reduced perfusion as a primary driver of abnormal *V*_A_/*Q* in CDH was confirmed by Weidner et al. who used magnetic resonance imaging to quantify lung perfusion in 2-year-old children following CDH repair and reported that pulmonary blood flow was reduced in the ipsilateral CDH lung.^20^ Our study is the first to report on the ventilation/perfusion relationships and degree of right-to-left shunt in the early postnatal period that encompasses birth, initial stabilisation, operation and weaning from invasive ventilation. We speculate that the initial low *V*_A_/*Q* in our cohort is explained by a more marked diffusion limitation/ventilation impairment compared to a relatively less abnormal pulmonary perfusion in the context of pulmonary hypoplasia. Furthermore, pulmonary hypertension in the immediate period following birth might also manifest with intracardiac right-to-left shunting which could not occur in the older children of the aforementioned follow-up studies, due to the functional closure of the foramen ovale and the ductus arteriosus shortly after birth. The finding of an improving *V*_A_/*Q* over the first days of life could be explained by lung expansion and improvement of ventilation, which is caused by invasive ventilation and the removal of the intestinal content from the thorax. Another potential explanation for the improving *V*_A_/*Q* might be the gradual reversal of hypoxic pulmonary vasoconstriction which would lead to diversion of blood from poorly ventilated to better-ventilated lung areas.

It should be noted that in absolute terms, the values of *V*_A_/*Q* that we are reporting are markedly abnormal with a median *V*_A_/*Q* of 0.20 in the whole cohort including survivors and non-survivors. These values are comparable to premature infants with severe bronchopulmonary dysplasia^21^ or pulmonary interstitial emphysema^22^ and more than four times lower than what would be seen in healthy term infants using the same methodology (median *V*_A_/*Q*: 0.84).^9, 10^ It was also interesting that although a median shunt of 10% was present in our population, the degree of shunt did not discriminate survivors from non-survivors, nor did it change significantly over the course of neonatal intensive care in the infants who survived to discharge. This might be explained by that right-to-left shunt is the result of pulmonary hypertension due to structural pulmonary blood vessel anomalies. The fixed/structural nature of these abnormalities might explain why the shunt could not be overcome during the initial period of neonatal stay.

We should also note that the nine infants who were excluded because of an unchanged oxygen requirement of 100% for their entire intensive care course, all died. Thus, our reported mortality refers only to the included infants, and the actual overall mortality for the study period is higher.

Compared to other predictors of mortality, the *V*_A_/*Q* demonstrated a high predictive ability with an area under the curve of 0.905. This is superior to the chest radiographic thoracic area we have previously reported which had an AUC of 0.826 and the LHR at diagnosis with an AUC of 0.698.^23^ We have, however, reported that the mean oxygenation index (OI) can predict mortality with 96% sensitivity and specificity.^24^ The better performance of the OI might be explained by the ability of the index to be applied in all infants including those nursed in 100% oxygen for the brief period before they die. Although most infants with CDH would have central arterial access in the first days after birth to calculate the OI, the non-invasive *V*_A_/*Q* offers the advantage that it can be calculated without invasive arterial sampling and at any time-point irrespective of the availability of arterial blood gas. Other methods to predict mortality in CDH include the size of the defect at the time of surgery and the need for a patch repair, which have both been associated with higher morbidity and decreased survival.^25, 26^ These predictors are limited, however, by that, they can only be applied in the infants who survive the surgery. Furthermore, other predictive measures of survival such as the Score for Neonatal Acute Physiology, Version II^27^ and the Score for Neonatal Acute Physiology Perinatal Extension,^28^ which are based on general physiological parameters (blood pressure, urine output, lowest temperature, seizures, birth weight and being small for gestational age), are not specifically designed to describe the pathophysiology of hypoxaemia in pulmonary hypoplasia and CDH. Our study presents a simple, non-invasive method that can specifically assess hypoxaemia and one which can predict mortality with moderate–high sensitivity.

One strength of our study is that it was performed in a single, high-volume centre that included a relatively severe population and a high incidence of the FETO procedure for which KCH is a referral centre. Another strength of our method is that the ODC method can directly assess the mechanisms of hypoxaemia (reduced *V*_A_/*Q* and increased right-to-left shunt) which is the main pathophysiological mechanism leading to mortality in CDH. Our conclusions refer to our population based on our local policies, but it would be of interest to establish whether our results could be replicated in a multicentre prospective study or in a different, less severe cohort in centres that do not routinely perform the FETO procedure. We should note that a limitation of our method was that it could not be applied in the severely unwell infants who were nursed in 100% oxygen throughout their brief stay and before they died. This group of infants however does not constitute a prognostic conundrum, as their anticipated course is clear very early in postnatal life. In our study, we used two retrospectively collected paired points of SpO_2_ and F_I_O_2_, whereas some previous neonatal studies have used 3–5 prospective pairs.^29, 30^ Two pairs, however, have also been used^9, 21, 22^ as the higher value of SpO_2_ would predominantly define the degree of shunt and the lower value of SpO_2_ would adequately define the *V*_A_/*Q*. The retrospective nature of our data, finally, might have left us with fewer paired measurements to calculate the *V*_A_/*Q* and shunt within our narrow time window of 4 h compared to prospectively collected data. The disease is however rare, and a prospective study of a similar number of infants would require a period of approximately ten years to complete, even in a high-volume centre.

In conclusion, we have described that the ventilation to perfusion relationship improved over the immediate postnatal period in surviving infants with congenital diaphragmatic hernia and non-invasive calculation of the ventilation to perfusion ratio in such infants can provide valuable information relating to the anticipated survival to discharge.
